# Clinical disease activity in autoimmune rheumatic patients receiving COVID-19 vaccines

**DOI:** 10.1186/s41927-024-00396-5

**Published:** 2024-06-17

**Authors:** Dzifa Dey, Bright Katso, Emmanuella Amoako, Aida Manu, Yaw Bediako

**Affiliations:** 1 Department of Medicine and Therapeutics, University of Ghana Medical School, Legon, Accra, Ghana; 2 Rheumatology Unit, Department of Medicine and Therapeutics, Korle-Bu Teaching Hospital, Accra, Ghana; 3 Yemaachi Biotechnology Ltd, Accra, Ghana

**Keywords:** COVID-19, Disease activity, Vaccination, Systemic lupus erythematosus (SLE), Rheumatoid arthritis (RA)

## Abstract

**Background:**

Vaccines are a crucial component of the global efforts to control the spread of COVID-19. Very little is known about COVID-19 vaccine responses in patients living with autoimmune rheumatic conditions in Africa. We examined the clinical reaction to COVID-19 vaccinations in Ghanaians diagnosed with autoimmune rheumatic disease.

**Methods:**

This was a hospital-based interventional cohort study of systemic lupus erythematosus (SLE) and rheumatoid arthritis (RA) patients recruited via regular face-to-face clinic visits. The systemic lupus erythematosus disease activity index Selena modification (SELENA-SLEDAI) and the disease activity score 28-joint count-erythrocyte sedimentation rate (DAS28-ESR) were used to measure changes in disease activity levels.

**Results:**

Thirty-eight (38) patients of which 21 (55.3%) were diagnosed with SLE and 17 (44.7%) with RA contributed data for analyses. Most (89.5%) of the patients were females, with a mean age of 37.4 years. The SLE patients experienced a notable increase in severe flares during weeks three and six, as well as the third and sixth months, followed by subsequent decreases in the twelfth month, while remission levels increased throughout the same period. Among RA patients, high disease activity decreased during weeks three and six, as well as the third, sixth, and twelfth months, with remission levels increasing during the same time. A low dose (≥ 50 < 75 mg) dose of azathioprine was at some point associated with having a severe flare among SLE patients. After both vaccine doses, SLE patients were the majority having experienced both local and systemic reactions, all resolving within 24 h. Approximately 73.7% of the patients were COVID-19 negative at baseline. During post-vaccination visits, this increased to 100% by week six, with no positives thereafter.

**Conclusion:**

This study explores COVID-19 vaccine responses in Ghanaian autoimmune rheumatic disease patients, revealing disease activity levels in RA patients improved after vaccination compared to SLE patients. Our findings identify a potential link between low-dose azathioprine and severe flares in SLE patients, particularly evident in the third-week post-vaccination. Further research is warranted to clarify these findings and guide tailored treatment approaches in this medically significant population during pandemics and vaccination efforts.

**Supplementary Information:**

The online version contains supplementary material available at 10.1186/s41927-024-00396-5.

## Key points

Disease activity increases in SLE patients at certain time points compared to RA patients after vaccination, indicating that vaccine responses vary by disease subtype and may influence disease outcome.

SLE patients who are undertreated (with Azathioprine) may experience severe flares following vaccination.

**Supplementary Information:**

The online version contains supplementary material available at 10.1186/s41927-024-00396-5.

## Introduction

By the last quarter of 2023, over 6.9 million people had died as a result of the coronavirus disease 2019 (COVID-19), and global public health, economic and social systems had been severely disrupted. Following the outbreak, vaccines became a crucial component of the global public health emergency efforts to control the spread of the disease [1]. In December 2020, the United Kingdom (UK) became the first country to approve the use of any of the COVID-19 vaccines, and billions of doses of these approved vaccines, including Pfizer-BioNTech, Moderna, and Oxford-AstraZeneca have been administered globally. According to OurWorldinData.org, about 70.6% of the world’s population has received at least one dose of a COVID-19 vaccine to date. By receiving 600,000 doses of the Oxford-AstraZeneca COVIShield vaccine (ChAdOx-nCoV1/AZD1222) in March 2021, Ghana became the first African nation to take advantage of the COVAX project [2, 3]. 

Despite the success of COVID-19 vaccines, concerns arose among those with autoimmune rheumatic diseases and taking immunosuppressive medications [4]. These immunosuppressive treatments often disrupt the immunological responses by vaccines, potentially diminishing vaccine effectiveness in these patient groups [5]. These concerns were compounded by the understanding that these patients face an elevated risk of severe acute respiratory syndrome coronavirus 2 (SARS-CoV-2) infection, severe COVID-19, and associated complications due to their underlying health conditions and medication regimens [6–11]. This further complicated vaccination decisions for individuals with autoimmune rheumatic diseases.

The COVID-19 vaccines have been found to be safe and effective in clinical trials and case-control studies involving healthy individuals [12–15]. However, during this study, little was known about the vaccine response in individuals with autoimmune rheumatic diseases and if they would mount an adequate immune response. Patients also expressed concerns about the vaccine’s potential impact on their immune system and whether it could trigger a flare-up of their condition [16, 17]. 

Though emerging evidence concerning the safety and efficacy of the vaccines in this category of patients is promising [18, 19], it is important to note that individual responses to the vaccine may vary based on the different vaccines, type and severity of the autoimmune disease as well as the medications used to manage the condition. Additionally, most of the earlier safety studies of COVID-19 vaccines among autoimmune rheumatic patients were in the Caucasian population and resided in high-income countries. Consequently, our understanding of how COVID-19 vaccines affect individuals with autoimmune diseases within the African population is limited. With the growing demand for information, especially as vaccines become more widely available, we seek to investigate the disease activity course after vaccination in Ghanaian autoimmune rheumatic patients.

## Materials and methods

### Study design and setting

We conducted a prospective hospital-based interventional cohort study at the Rheumatology Unit, Department of Medicine and Therapeutics, Korle Bu Teaching Hospital (KBTH) from January to December 2022. Being one of the physician specialist units in the hospital, the Rheumatology Unit provides outpatient and inpatient services to patients with a wide variety of autoimmune rheumatic diseases. Patients seeking specialised care at this unit travel from various parts of Ghana and nearby West African countries. Participants were recruited via regular face-to-face visits to the rheumatology clinic and scheduled again at one week, three weeks, six weeks, three months, six months, and twelve months for post-recruitment visits. Vaccines administered were Oxford-AstraZeneca and Pfizer-BioNTech. Each participant received two doses, three weeks between 1st and 2nd doses for those who received Pfizer-BioNTech and six weeks between 1st and 2nd doses for Oxford-AstraZeneca.

### Inclusion and exclusion criteria

Participants included in the study were adult patients aged ≥ 18 of self-reported African lineage meeting American College of Rheumatology (ACR) or European League Against Rheumatism EULAR criteria for a diagnosis of systemic lupus erythematosus (SLE) or rheumatoid arthritis (RA), and enrolled in the clinic’s register either before or during the pandemic. Exclusion criteria encompassed patients with prior COVID-19 vaccination, documented allergies or contraindications to COVID-19 vaccines, those presenting with highly active disease at the study’s onset, and individuals unable to provide consent.

### Safety evaluations

Detailed safety examinations were conducted throughout the study. These included COVID-19 positivity, full blood count, urinalysis, blood urea and electrolytes, c-reactive protein, erythrocyte sedimentation rate, and complement levels.

### Outcomes

Patient demographics, baseline clinical characteristics, and anthropometric measures were recorded. Patient-reported outcomes included the SELENA-SLEDAI [20], the Disease Activity Score 28- erythrocyte sedimentation rate (DAS28-ESR) [21], and other reported clinical disease features assessed by a study-specific questionnaire, which is provided as supplementary material. Laboratory outcomes from safety evaluations were populated into the tools to estimate the SELENA-SLEDAI and DAS28-ESR scores appropriately. The instruments were evaluated by a trained rheumatologist with previous experience evaluating SLE disease activity parameters and more than ten years of experience managing patients with various kinds of autoimmune diseases.

### Statistical analysis

Descriptive statistics were employed to present participant characteristics and various study variables. Disease activity measured by the SELENA-SLEDAI and DAS28-ESR scores was assessed prospectively during subsequent visits. Disease activity status was defined as follows: For SELENA-SLEDAI; Remission [SLEDAI = 0 points], Mild to Moderate flare/disease activity [SLEDAI ≥ 3 points to ≤ 12 points and a ≥ 1-point increase in PGA], and Severe flare/disease activity [SLEDIA > 12 points and a ≥ 2.5 increase in PGA]. For DAS28-ESR; Complete remission [score < 2.6], Low-Moderate disease activity [score ≥ 2.6 to < 3.2 - score ≥ 3.2 to ≤ 5.1], High disease activity [score > 5.1]. All analyses encompassed data for the baseline and subsequent visits except for the first week. Week one visits were excluded from the analysis as they occurred too soon after the baseline visit to capture significant changes in disease activity. The chi-square or Fisher’s exact test as appropriate estimated any association between study variables (disease activity changes) and (demographic and clinic variables) at baseline and consecutive visits. Additionally, correlation analysis evaluates the degree of association between various categories of medication dosage and disease activity levels.

### Ethical consideration

The current study sought approval from the Korle Bu Teaching Hospital’s Institutional Review Board (IRB) which is made up of two committees; the Scientific and Technical Committee (STC) and the Ethics Committee. Approval was granted under the number *KBTHSTC 000195/2021.* Participants received information about the study objectives, their rights, the potential benefits and risks associated with participation, and the measures taken to safeguard their privacy. Each participant then voluntarily and knowingly provided written consent to partake in the study. To maintain the confidentiality of data, unique identification codes were assigned to each individual.

## Results

This study initially recruited forty (40) patients, but two were discontinued after baseline due to the exclusion criteria. The final analysis included 38 patients with at least four continuous visits per patient. Table 1 shows a female dominance of the participants (89.5%), with over half (55.3%) of them diagnosed with Systemic Lupus Erythematosus (SLE). Among the patients diagnosed with Rheumatoid Arthritis (RA), the mean ± SD age was 43.4 ± 9.6 years, while SLE patients had a mean ± SD age of 32.5 ± 8.9 years. The RA patients also exhibited a higher mean Body Mass Index (BMI) of 33.4 ± 10.1, in contrast to the SLE patients, who had a BMI of 22.6 ± 7.5. The total number of patients who received the first and second doses of the AstraZeneca vaccine was 63.2%, while 36.8% received the BioNTech and Pfizer vaccines.

**Table 1:** Participants’ bassline demographic and clinical characteristics

Characteristics	Total Patients (*n* = 38)	SLE (*n* = 21)	RA (*n* = 17)
**Age, mean ± SD years**	37.4 ± 10.6	32.5 ± 8.9	43.4 ± 9.6
**Sex**			
Female	34 (89.5)	20 (58.8)	14 (41.2)
Male	4 (10.5)	1 (25.0)	3 (75.0)
**Body Mass Index (BMI) kg/m** ^**2**^	29.7 ± 9.3	22.6 ± 7.5	33.4 ± 10.1
**Disease Duration, mean ± SD years**	2.8 ± 0.8	2.8 ± 0.7	2.6 ± 0.8
**Comorbidities**			
Hypertension	4 (10.5)	2 (9.5)	2 (11.8)
Kidney disease	2 (5.3)	2 (9.5)	0 (0)
Asthma	1 (2.6)	1 (4.8)	0 (0)
Diabetes	2 (5.3)	0 (0)	2 (11.8)
**Medications**			
Prednisolone	37 (97.4)	21 (100)	16 (94.1)
*Low(≤ 10 mg)*	*8 (21.6)*	*4 (19.0)*	*4 (25.0)*
*Moderate (> 10 ≤ 20 mg)*	*27 (73.0)*	*15 (71.4)*	*12 (75.0)*
*High (> 20 mg)*	*2 (5.4)*	*2 (9.5)*	*0 (0.0)*
Hydroxychloroquine	35 (92.1)	21 (100)	14 (82.4)
*Low(200 mg)*	*6 (17.1)*	*3 (14.3)*	*3 (21.4)*
*High (400 mg)*	*29 (82.9)*	*18 (85.7)*	*11 (78.6)*
Azathioprine	12 (31.6)	12 (57.1)	0 (0)
*Low (≥ 50 < 75 mg)*	*4 (33.3)*	*4 (33.3)*	*0 (0.0)*
*Moderate (≥ 75 < 100 mg)*	*4 (33.3)*	4 (33.3)	*0 (0.0)*
*High (≥ 100 mg)*	*4 (33.3)*	4 (33.3)	*0 (0.0)*
Methotrexate	10 (26.3)	2 (9.5)	8 (47.1)
*Low (≤ 7.5 mg)*	*5 (50.0)*	*0 (0.0)*	*5 (62.5)*
*Moderate (> 7.5 ≤ 15 mg)*	*2 (20.0)*	*0 (0.0)*	*2 (25.0)*
*High (> 15 mg)*	*3 (30.0)*	*2 (100.0)*	*1 (12.5)*
Sulfasalazine	2 (5.3)	0 (0)	2 (11.8)
Mycophenolate mofetil	2 (5.3)	2 (9.5)	0 (0)
Omeprazole	31 (81.6)	17 (80.9)	14 (82.3)
Calcium	31 (81.6)	17 (80.9)	14 (82.3)
Others (e.g., folic acid)	23 (60.5)	8 (38.1)	15 (88.2)
**COVID-19 Vaccines**			
Oxford/AstraZeneca (1&2 doses)	24 (63.2)	14 (66.7)	10 (58.8)
BioNTech, Pfizer vaccine (1&2 doses)	14 (36.8)	7 (33.3)	7 (41.2)

No cases of mortality were recorded during the study. The average disease duration was 2.7 ± 0.7 years, with 44.7% of patients having the disease for five years, 34.2% for 6–10 years, and 21.1% for more than ten years. The majority of the patients were on Prednisolone (94.7%), Hydroxychloroquine (92%), and also both Omeprazole and Calcium (81.6%). Prednisolone was most commonly prescribed at a dosage of 10 mg and hydroxychloroquine at 400 mg. A higher percentage of SLE patients (62.1%) were on the higher dose of hydroxychloroquine i.e. (400 mg) compared to RA patients (37.9%) on this dosage. Similarly, the highest dose of prednisolone (40 mg) was prescribed to SLE patients, while RA patients received a maximum dose of 20 mg. Additional immunosuppressants included Azathioprine (31.6%), Methotrexate (26.3%), Mycophenolate Mofetil (5.3%), and Sulfasalazine (5.3%).

Following the first dose of the vaccines, local reactions were documented, including severe pain at the injection site in 22 out of the total 38 patients, with the majority (53.3%) being SLE patients. Swelling at the injection site was observed in 6 patients, and the majority (66.7%) were SLE patients. Systemic reactions, including chest pain/tightness/breathlessness, were documented in 3 patients, all of whom were SLE cases. Headaches were reported by 12 patients, mostly (83.3%) by SLE patients. Following the second dose of the vaccines, local reactions included severe pain at the injection site reported in 5 patients, the majority (60%) of whom were SLE cases, and swelling of the site reported by two patients, all being SLE cases. Headaches were also reported after second doses in 5 patients, 83.3% of whom were SLE patients, while other symptoms such as body itches and sweat were reported by 6 patients, most of whom were SLE patients. The mean ± SD time for reaction onset after the first dose was 26.0 ± 16.2 h, reported among the patients. Following the second dose, however, this duration increased to 29.2 ± 16.5 h. All the local and systemic reactions were resolved without any patient requiring hospitalisation or medications. Details are shown in Table 2.

**Table 2:** Patient reactions post first and second COVID-19 vaccine doses

Following Dose 1			
	(*n* = 38)	SLE	RA
**Mean ± SD time (hrs) for reaction onset**	26.0 ± 16.2		
Swelling at the site	6	4(66.7)	2 (33.3)
Abscess formation at the site	0	0 (0.0)	0 (0.0)
Severe pain at the site	15	8 (53.3)	7 (46.7)
Fever/chills	6	5 (83.3)	1 (16.7)
Allergies	0	0 (0.0)	0 (0.0)
Diarrhoea	1	1 (100)	0 (0.0)
Nausea/vomiting	2	2 (100)	0 (0.0)
Seizures	0	0 (0.0)	0 (0.0)
Tingling sensation/numbness in the limbs	1	0 (0.0)	1 (100)
Weakness in any part of the body	7	3 (42.9)	4 (57.1)
Headaches	12	10 (83.3)	2 (16.7)
Cytopenia	0	0 (0.0)	0 (0.0)
Chest pain/tightness/breathlessness	3	3 (100)	0 (0.0)
Arthralgia/arthritis	2	0 (0.0)	2 (100)
Others, e.g., body itches, sweating	4	1 (25.0)	3 (75.0)
Hospitalisations due to reaction	0	0 (0.0)	0 (0.0)
**Following Dose 2**			
**Mean ± SD time (hrs) for reaction onset** 29.2 ± 16.5			
Swelling at the site	2	2 (100)	0 (0.0)
Abscess formation at the site	1	0 (0.0)	1 (100)
Severe pain at the site	5	3 (60.0)	2 (40.0)
Fever/chills	2	2 (100)	0 (0.0)
Allergies	2	2 (100)	0 (0.0)
Diarrhoea	0	0 (0.0)	0 (0.0)
Nausea/vomiting	1	0 (0.0)	1 (100)
Seizures	0	0 (0.0)	0 (0.0)
Tingling sensation/numbness in the limbs	1	0 (0.0)	1 (100)
Weakness in any part of the body	3	1 (33.3)	2 (66.7)
Headaches	6	5 (83.3)	1 (16.7)
Cytopenia	0	0 (0.0)	0 (0.0)
Chest pain/tightness/breathlessness	4	2 (50.0)	2 (50.0)
Arthralgia/arthritis	0	0 (0.0)	0 (0.0)
Others, e.g., body itches, sweating	6	4 (66.7)	2 (33.3)
Hospitalisations due to reaction	0 (0.0)	0 (0.0)	0 (0.0)

For the SLE patients, mild to moderate flares/disease activity was defined as SLEDAI scores of ≥ 3 points to ≤ 12 points and a corresponding increase in Physician Global Assessment (PGA) score of at least 1 point. Severe flares were defined as SLEDAI scores exceeding 12 points accompanied by a PGA score increase of at least 2.5 points. Remission was indicated by a SLEDAI score of 0 points. Table 3 shows the mean ± SD SLEDAI score at baseline to be 7.1 ± 5.5. At this baseline visit, approximately 52.4% of the SLE patients were classified as experiencing mild to moderate disease activity, 28.6% were classified as experiencing severe disease activity, and 19% were classified as being in remission.

During follow-up visits, the percentage of SLE patients experiencing mild to moderate flares decreased consistently compared to the baseline measurement. Reductions of 9.6%, 23.8%, 23.8%, and 19.1% were observed at weeks three and six as well as the third and sixth months, respectively. However, in the twelfth month, mild to moderate flares increased by 10.6% compared to the baseline’s 52.4%, resulting in 63% of the SLE patients having experienced mild to moderate flares at month 12.

On the other hand, SLE patients experiencing severe flares during follow-up visits consistently increased by 4.8%, 9.5%, 20%, and 9.5% at weeks three and six, as well as the third and sixth months, respectively, relative to the baseline. However, they decreased by 7.5% in the twelfth month.

Remission levels among the patients during follow-up visits showed increases of 4.8%, 14.3%, 4.8%, and 9.6% at weeks three and six, as well as the third and sixth months, respectively, compared to the baseline. However, it decreased by 3.2% in the twelfth month, relative to the baseline.

**Table 3:** Change in disease activity - SLEDAI score over the study period

	Mild- Moderate flare	Severe flare	Remission	Total (*n*)
*Baseline*	11 (52.4)	6(28.6)	4(19.0)	21
*3 Weeks*	9 (42.8)	7 (33.4)	5 (23.8)	21
*6 Weeks*	6 (28.6)	8 (38.1)	7 (33.4)	21
*3 Months*	6 (28.6)	10 (47.6)	5 (23.8)	21
*6 Months*	7 (33.3)	8 (38.1)	6 (28.6)	21
*12 Months*	12 (63.0)	4 (21.1)	3 (15.8)	19

Significant disease activity changes were observed among the SLE patients throughout the study. Except for week three, patient demographic and clinical characteristics were not found to be significantly associated with these observed disease activity changes. During the week three visit, azathioprine showed a significant association with the changes in disease activity levels observed, indicated by a *p*-value of *0.03*, as shown in Table 4. This was explored further whereby the dosages of azathioprine in this study were categorised as low dose (between 50 and 75 mg), moderate dose (between 75 and 100 mg), high dose (between 100 and 125 mg), and very high dose (above 125 mg). Specifically, a significant correlation was observed between experiencing a severe flare and being on a low dose of azathioprine (*r* = 1, *p* = 0.05).

**Table 4:** Association between patient characteristics and disease activity at week three

	SLEDAI-Level *n* = 21 (%)	*P*-Value	DAS-Level *n* = 17 (%)	*P*-Value
Age	21 (100.0)	0.38	17 (100.0)	0.69
Gender-*female*	20 (95.2)	0.39	14 (82.4)	0.84
Hydroxychloroquine *- yes*	21 (100.0)	0.37	14 (82.4)	0.30
Prednisolone- *yes*	21 (100.0)	0.38	16 (94.1)	0.13
Methotrexate - *yes*	2 (9.5)	0.09	8 (47.1)	0.17
Mycophenolate mofetil - *yes*	2 (9.5)	0.42	0 (0.0)	
Azathioprine- *yes*	12 (57.1)	0.03*	1 (5.9)	0.78
Sulfasalazine - *yes*	0 (0.0)		2 (11.8)	0.85

Among the RA patients, complete remission was indicated by a DAS28-ESR score of less than 2.6, low to moderate disease activity was defined as a DAS28-ESR score of 2.6 to less than 5.1, while high disease activity was characterised by a DAS28-ESR score exceeding 5.1. Table 5 shows the mean ± SD DAS28-ESR score at baseline to be 4.0 ± 1.2. At this baseline visit, approximately 70.6% of the RA patients exhibited low to moderate disease activity, with an additional 17.6% experiencing high disease activity and 11.8% in remission.

During the follow-up visits, the percentage of RA patients experiencing low to moderate disease activity consistently decreased by 5.9%, 5.9%, and 1.8% at weeks three and six, as well as the third month, respectively, compared to the baseline proportion of 70.6%. However, it increased by 4.4% and 10.7% in the sixth and twelfth months respectively, relative to the baseline.

High disease activity among the RA patients during follow-up visits saw a decline of 11.7%, 5.8%, and 11.3% at the third and sixth-week visits, as well as the third-month visit, relative to the baseline. Subsequently, the percentage of RA patients that experienced high disease decreased to 0% compared to the baseline during the sixth and twelfth-month visits.

Conversely, remission levels among the RA patients increased by 17.6%, 11.7%, 13.2%, 13.2%, and 7% at weeks three and six as well as the third, sixth, and twelfth months, respectively, relative to the baseline measurement.

**Table 5:** Change in disease activity – DAS28 score over the study period

	Low-Moderate	High disease	Remission	Total (*n*)
*Baseline*	12 (70.6)	3 (17.6)	1 (11.8)	17
*3 Weeks*	11 (64.7)	1 (5.9)	5 (29.4)	17
*6 Weeks*	11 (64.7)	2 (11.8)	4 (23.5)	17
*3 Months*	11 (68.8)	1 (6.3)	4 (25.0)	16
*6 Months*	12 (75.0)	0 (0.0)	4 (25.0)	16
*12 Months*	13 (81.3)	0 (0)	3 (18.8)	16

Unlike the SLE patients, no association was established between the study variable (change in disease activity) and the various demographic or clinical characteristics among the RA patients.

Table 6 summarises trends in PCR test results for COVID-19 positivity. At baseline, 73.7% of patients tested negative for COVID-19, while 26.3% tested positive. Following vaccination, the percentage of patients testing negative consistently increased over time, reaching 100% by week six and remaining at 100% throughout the subsequent follow-up periods. The percentage of patients testing positive for COVID-19 decreased rapidly after vaccination, with only 2 (5.3%) positive cases observed in the first week, 1 (2.6%) positive case during the third week and thereafter, no patient tested positive for COVID-19 throughout the subsequent follow-up visits.

**Table 6:** COVID-19 PCR positivity among the patients throughout the study

	Follow-up period						
	Baseline	Week 1	Week 3	Week 6	Week 12	Month 6	Month 12
	*N* = 38	*N* = 38	*N* = 38	*N* = 38	*N* = 37	*N* = 37	*N* = 35
	*n*/*N* (%)	*n*/*N* (%)	*n*/*N* (%)	*n*/*N* (%)	*n*/*N* (%)	*n*/*N* (%)	*n*/*N* (%)
**COVID-19 PCR test results**							
**Negative**	28/38 (73.7%)	36/38 (94.7%)	37/38 (97.4%)	38/38 (100.0%)	37/37 (100.0%)	37/37 (100.0%)	35/35 (100.0%)
**Positive**	10/38 (26.3%)	2/38 (5.3%)	1/38 (2.6%)	0/38 (0.0%)	0/38 (0.0%)	0/38 (0.0%)	0/38 (0.0%)

## Discussion

This study aimed to assess the changes in clinical disease activity among autoimmune rheumatic disease patients in Ghana following their COVID-19 vaccination. Research data on vaccine responses are crucial in this pandemic era to shape, and direct vaccination programs worldwide, even more so in chronic conditions such as autoimmune rheumatic diseases where individuals have compromised immunity and concerns about vaccine response. Addressing issues surrounding vaccine responses in this patient population, the need for additional studies and research data spanning diverse geographical and ethnic backgrounds adds to corroborate the overall COVID-19 vaccine response in these patients.

Our study presents findings on disease activity changes over time for SLE and RA patients after COVID-19 vaccination. Notably, it reveals a consistent increase in severe flares among SLE cases, accompanied by an increase in remission levels. Conversely, RA patients experienced a progressive decrease in high disease activity and increased remission levels. Previous studies investigating post-vaccination responses in autoimmune rheumatic conditions primarily focused on assessing antibody levels targeting the SARS-COV-2 Spike protein in these patients, often in comparison to healthy populations [22]. Additionally, some studies concentrated on evaluating reactogenicity and characterising the local and systemic reactions to COVID-19 vaccines, comparing data from these patient groups with data from healthy populations [23–26]. Notably, these multi-centre studies extended beyond SLE and RA but lacked information about the disease states of the various autoimmune conditions examined before and after vaccination, a unique aspect which is covered in our study.

Our study detected significant changes in disease activity levels post-vaccination compared to pre-vaccination or baseline levels. A similar observation was made by van Dam et al. in a large cohort study focusing on immune-compromised patients following SARS-COV-2 vaccinations. Their study which shared similarities with ours in terms of design, vaccine range, and timing, reported a 10% increase in disease activity after primary vaccination and an 11% increase after the first additional vaccination [27]. Unlike the current study that focused on specific immune-mediated inflammatory diseases, that of van Dam et al. was generalised. All categories of disease activity in both disease groups in our study experienced a change after vaccination. In contrast, two observational analyses involving cohorts of autoimmune inflammatory rheumatic disease patients found that disease activity remained relatively stable in the majority of patients after COVID-19 vaccinations [18, 19]. Additionally, a large cohort study involving patients with rheumatoid arthritis and psoriatic arthritis reported no substantial changes in disease activity levels following COVID-19 vaccination, unlike this study [28]. 

This study identified an increase in severe flares among SLE patients from week three through to the sixth month following COVID-19 vaccination. Except for azathioprine, none of the demographic and clinical variables exhibited an association with the observed changes in disease activity throughout this period. Low-dose azathioprine displayed a strong positive correlation with severe flares among SLE patients. This may be due to decreased dosage post-vaccination, which was advised during the pandemic’s early stages or patients who had uncontrolled disease due to suboptimal dosage of therapy would be more likely to experience flares.

Rheumatoid Arthritis (RA) patients exhibited a more substantial improvement in disease activity compared to Systemic Lupus Erythematosus (SLE) patients throughout the study period. Notably, there was a rapid decline in high disease activity levels within the first three weeks, and this trend continued up to the third-month visit. Furthermore, during the subsequent sixth- and twelfth-month visits following vaccination, none of the RA patients experienced high disease activity. These findings hold significance in understanding how different patient subgroups respond to vaccines, which may inform tailored vaccination strategies based on specific disease characteristics. Our study also revealed a significant decrease in local and systemic reactions following the first COVID-19 vaccine dose compared to earlier studies involving similar patient populations [23, 24]. Notably, the majority of these reactions fell into the mild category and resolved without any hospitalisations, underscoring the safety and tolerability of these vaccines among patients with RA and SLE. The mean time for reaction onset after the second dose in our study is slightly higher (29.2 h) compared to after the first dose (26.0 h). The increase in mean onset time from the first to the second dose could potentially indicate a gradual strengthening of the immune response with subsequent doses. This is a common phenomenon observed with vaccines, where the immune system becomes more adept at recognising and responding to the antigen with each exposure [29]. Moreover, findings from the current study demonstrate a clear trend of increasing COVID-19 negativity and decreasing positivity following vaccination. Vaccination appears to be highly effective in preventing COVID-19 infection, as evidenced by the absence of positive PCR tests in the later follow-up periods. The earlier positive tests may indicate improved protection over time.

While our study was not designed as a vaccine efficacy assessment, it substantially supports a favourable risk-benefit ratio over an extended period, contributing further to the safety profile of COVID-19 vaccines for autoimmune rheumatic disease patients, as documented by earlier studies [18, 19]. It provides valuable data regarding the trajectory of disease activity following vaccination in distinct autoimmune diseases inadequately explored in the context of COVID-19 vaccines. We observed a gradual increase in severe flares among SLE patients, although the corresponding rise in remission levels, while notable, did not reach a comparable magnitude. Moreover, the improvement in disease activity observed among RA patients and a concurrent increase in remission levels remained consistent throughout the study period.

It is important to mention that, throughout the study period, no major changes or adjustments were recorded or made to patients’ medications. This consistency in treatment regimens allowed us to minimise the potential confounding effects of treatment changes on the observed disease activity levels. Moreover, we conducted association tests to explore the relationship between treatments and changes in disease activity levels. Together, we believe this comprehensive approach has addressed any concern regarding the potential impact of treatment changes on our study outcomes.

Our study encounters some limitations, mainly the relatively small sample size, comprising 38 patients. A case-control design, facilitating a comparative analysis of disease activity changes, could provide better insights into the vaccination’s impact on disease activity. However, these challenges arise from the fact that a significant portion of the patient cohort had already received one or both doses of the COVID-19 vaccine before the study commenced due to the effective vaccine education efforts. Including these pre-vaccinated patients in the analysis would introduce irregularities and undermine the accuracy of measuring or matching disease activity changes over the study period.

## Conclusion

This study provides valuable insights into COVID-19 vaccine responses among Ghanaians diagnosed with autoimmune rheumatic diseases. Vaccine responses in these patients exhibit variability based on the specific disease subtype, which may impact disease outcomes. The study revealed a notable correlation between low-dose azathioprine and severe flares among SLE patients, particularly evident at the week three visit post-vaccination. Even though this association was not consistently observed across subsequent study visits, it suggests a potential link between reduced azathioprine dosage and vaccination-related flares in SLE patients, warranting further investigation to better understand the underlying mechanisms and implications for clinical management. This nuanced understanding could inform personalised treatment strategies and optimise outcomes in this often underrepresented yet medically significant patient population, especially amidst pandemics and vaccination scenarios.

### Electronic supplementary material

Below is the link to the electronic supplementary material.

Supplementary Material 1

## Data Availability

The datasets analysed during the current study are available from the corresponding author on reasonable request.

## Abbreviations

ACR: American College of Rheumatology
COVID-19: Coronavirus Disease 2019
DAS28-ESR: Disease Activity Score28-Joint Count-Erythrocyte Sedimentation Rate
EULAR: European Alliance of Associations for Rheumatology
KBTH: Korle Bu Teaching Hospital
Kg: Kilogram
Mg: Milligram
RA: Rheumatoid arthritis
SARS-CoV-2: Severe acute respiratory syndrome coronavirus 2
SD: Standard Deviation
SLE: Systemic Lupus Erythematosus
SLEDAI: Systemic Lupus Erythematosus Disease Activity Index
UK: United Kingdom
