# Supplementary material for: Clinical disease activity in autoimmune rheumatic patients receiving COVID-19 vaccines
Source: BMC Rheumatol. 2024 Jun 17;8:25. doi: 10.1186/s41927-024-00396-5 (PMC11181637; doi:10.1186/s41927-024-00396-5)
Supplement: Supplementary file 1 — Supplementary Material 1 [file 41927_2024_396_MOESM1_ESM.docx]

**INFORMATION FORM**

This form is being given to you because you are being identified as a potential subject for an ongoing research study. The sections below provide general information about the study and your rights as a potential participant. The research staff will take you through the information. Feel free to ask questions at any time. It is your choice to take part in this study by giving your consent. Be assured there will be no consequences whatsoever if you do not take part in this study.

The purpose of this study is to investigate the trajectory of disease activity after COVID-19 vaccination in Ghanaian autoimmune rheumatic patients receiving care at the Korle Bu Teaching Hospital. This study will be a prospective cohort study and we plan to engage participants again after 1 week, 3 weeks, 6 weeks, 3 months, 6 months and 12 months post-recruitment. We anticipate that each of these sections should not exceed 20 minutes.

Your participation in this study may bring some minor risks and discomforts. You are likely to experience pain, dizziness, soreness, skin irritation or rash, minor bruising and bleeding into the skin. Participants should also expect minor discomfort following full observance of COVID-19 protocols.

Your participation in this study will help generate important insights into the overall understanding of how COVID-19 vaccines affect individuals with autoimmune diseases within the African population, a knowledge that is currently limited. By directly studying vaccine responses among Ghanaians, this study will also provide the opportunity to clear the doubts and reservations among Ghanaian adults concerning the COVID-19 vaccines and hopefully lower the level of hesitancy among the population, especially the vulnerable population of persons with autoimmune rheumatic conditions.

Your health and any other information gathered will remain private and confidential and will only be accessible to the research team. You will be assigned an ID number to avoid tracing specific responses to your name.

Full participation is essential to achieving the outcome of this study. Nevertheless, participation is voluntary and you are at liberty to pull out from the study at any point with no repercussions.

No benefit in the form of cash or food will come to you during and after your participation in this study. Similarly, the researcher will ensure you do not incur any risk because you participated in this study. A copy of the Information sheet will be given to you after it has been signed to take home.

**Contact for additional information.**

Should you need additional information and clarification on this study, or in case you incur an injury in the course of this study, please contact;

DR DZIFA DEY of the Department of Medicine, University of Ghana Medical School, KBTH on: **0244672343** or [**dzifakay@gmail.com**](mailto:dzifakay@gmail.com)

If you have any ethical concerns as a participant in this study, kindly contact the KBTH research unit.

CONSENT FORM

The above document describing the purpose, benefits, risks and procedures for the research project; **Clinical Disease Activity in Autoimmune Rheumatic Patients Receiving COVID-19 Vaccines** has been read and explained to me in detail. I understand a copy of the information form and Consent statement will be giving to me to take home after signing. I have been allowed to ask questions about the research and they were all answered to my satisfaction. I agree to participate in this research study as a volunteer.

______________________________ _______________________ ___________

Name of Participant: Signature: Date:

**Witness**

I was present while the above document detailing benefits, risks and procedures for the study were read to the volunteer. All questions were answered and the volunteer has agreed to take part in the research.

________________________________ ______________________ ____________

Name: Signature of Witness: Date:

**Obtaining Consent**

I have fully explained the nature, the risk and the purpose of this study to the participants. I have answered and will answer to the best of my ability all questions related to this study.

_______________________________ __________________ ______________

Name: Signature: Date

BASELINE QUESTIONNAIRE

FIRST VISIT

*Required

1. Participant ID

H-Site code- 0000

1. Date

*Example: 7 January 2022*

1. Vaccination site
2. Name of participant
3. Phone number

Required digits

1. Sex

*Mark only one oval.*

Female

Male

1. Age *
2. Height *
3. Weight *
4. Which Autoimmune condition were you diagnosed with? please specify.
5. Which year were you diagnosed?
6. Are you on any medication for the above illness?

*Mark only one oval.*

Yes
No

1. if yes, list medication(s)
2. Have you been diagnosed with any of the following underlying conditions? [check all that apply]

Other:

*Tick all that apply.*

Heart Disease/ Hypertension

Kidney Disease

Asthma

TB

Epilepsy

Diabetes

Jaundice or Hepatitis

HIV

1. What vaccine are you receiving today?
2. Batch Number
3. Name of Vaccine

*Mark only one oval.*

AstraZeneca

Pfizer

FOLLOW-UP QUESTIONNAIRE

1. Date

*Example: 7 January 2022*

1. Participant ID H-Site code- 000

# Swelling

3. Did you have any swelling at the vaccine site?

*Mark only one oval.*

Yes

No *Skip to question 6*

# Swelling 2

1. When did it appear?

*Mark only one oval.*

<24 hours

24-72 hours

>72 hours

1. Outcome

*Mark only one oval.*

Resolved

Ongoing

# Abscess formation

6. Did you have abscess /boil formation at the vaccine site?

*Mark only one oval.*

Yes

No *Skip to question 9*

# Abscess 2

1. When did it appear?

*Mark only one oval.*

<24 hours

24-72 hours

>72 hours

1. Outcome

*Mark only one oval.*

Resolved

Ongoing

# Pain

9. Did you have severe pain at the vaccine site?

*Mark only one oval.*

Yes

No *Skip to question 12*

# Pain 2

1. When did it occur?

*Mark only one oval.*

< 24 hrs

24 -- 72 hrs

> 72 hrs

1. Outcome

*Mark only one oval.*

Resolved

Ongoing

# Fever

12. Did you experience any fever or chills after vaccination?

*Mark only one oval.*

Yes

No *Skip to question 15*

# Fever 2

1. When did it start?

*Mark only one oval.*

<24 hours

24-72 hours

>72 hours

1. Outcome

*Mark only one oval.*

Resolved

Ongoing

# Allergic reaction

15. Did you experience any allergic reaction from the vaccination?

*Mark only one oval.*

Yes

No *Skip to question 19*

# Allergic reaction 2

1. What type of allergy?

*Mark only one oval.*

Rash

Anaphylaxis

Other:

1. When did it occur? *Mark only one oval.*

< 24 hrs

24 -- 72 hrs

> 72 hrs

1. Outcome

*Mark only one oval.*

Resolved

On going

# Diarrhea

19. Did you have any bout of diarrhea after vaccination?

*Mark only one oval.*

Yes

No *Skip to question 22*

Other:

# Diarrhea 2

1. When did it start?

*Mark only one oval.*

< 24 hrs

24 -- 72 hrs

> 72 hrs

1. Outcome

*Mark only one oval.*

Resolved

On going

# Nausea/Vomiting

22. Did you experience any nausea/vomiting post vaccination?

*Mark only one oval.*

Yes

No *Skip to question 25*

# Nausea/Vomiting 2

1. When did it start?

*Mark only one oval.*

< 24 hrs

24 -- 72 hrs

> 72 hrs

1. Outcome

*Mark only one oval.*

Resolved

On going

# Chest Pain

25. Did you experience any chest pain or chest tightness/breathlessness post vaccination?

*Mark only one oval.*

Yes

No *Skip to question 28*

# Chest Pain 2

1. When did it start?

*Mark only one oval.*

< 24 hrs

24 -- 72 hrs

> 72 hrs

1. Outcome

*Mark only one oval.*

Resolved

On going

# Seizures

28. Did you have any seizures after vaccination?

*Mark only one oval.*

Yes

No *Skip to question 31*

# Seizures 2

1. When did it start?

*Mark only one oval.*

< 24 hrs

24 -- 72 hrs

> 72 hrs

1. Outcome

*Mark only one oval.*

Resolved

On going

# Tingling sensation/Numbness

31. Did you experience any tingling sensation/numbness in any limbs?

*Mark only one oval.*

Yes

No *Skip to question 34*

# Tingling sensation/Numbness 2

1. When did it start?

*Mark only one oval.*

< 24 hrs

24 -- 72 hrs

> 72 hrs

1. Outcome

*Mark only one oval.*

Resolved

On going

# Weakness 1

34. Did you feel weakness in any part of the body?

*Mark only one oval.*

Yes

No *Skip to question 38*

# Weakness 2

1. Specify:
2. When did it start?

*Mark only one oval.*

< 24 hrs

24 -- 72 hrs

> 72 hrs

1. Outcome

*Mark only one oval.*

Resolved

On going

# Headaches

38. Did you experience any headaches after vaccination?

*Mark only one oval.*

Yes

No *Skip to question 41*

# Headaches 2

1. When did it start?

*Mark only one oval.*

< 24 hrs

24 -- 72 hrs

> 72 hrs

1. Outcome

*Mark only one oval.*

Resolved

On going

# Blood Problem

41. Did you experience any blood problems?

*Mark only one oval.*

Yes

No *Skip to question 45*

# Blood Problem 2

1. Specify which blood problems you had:
2. When did it start?

*Mark only one oval.*

Yes

No

1. Outcome

*Mark only one oval.*

Resolved

On going

# Joint pains

1. Did you experience any headaches after vaccination?

*Mark only one oval.*

Yes

No *Skip to question 48*

# Joint pains 2

1. When did it start?

*Mark only one oval.*

< 24 hrs

24 -- 72 hrs

> 72 hrs

1. Outcome

*Mark only one oval.*

Resolved

On going

# Other Side Effects

1. Did you experience any other side effect that was not mentioned above?

*Mark only one oval.*

Yes

No *Skip to question 52*

# Other Side Effects 2

1. Specify:
2. When did it start?

*Mark only one oval.*

< 24 hrs after vaccine

24 -- 72 hrs after vaccine

> 72 hrs

1. Outcome

*Mark only one oval.*

Resolved

On going

# Medication

1. Did you need to take medication to help with any side effects?

*Mark only one oval.*

Yes

No *Skip to question 54*

# Medication 2

1. Specify which medicine(s) you took for any of your side effects:

# Hospitalisation

1. Did you require hospitalisation for any side effects?

*Mark only one oval.*

Yes

No

# Hospitalisation 2

1. How long were you admitted for (in days)?
2. Were you diagnosed with any condition related (or unrelated) to the vaccine at the hospital?

*Mark only one oval.*

Yes

No

1. What are the conditions?
